# Tension-driven axon assembly: a possible mechanism

**DOI:** 10.3389/fncel.2015.00316

**Published:** 2015-08-12

**Authors:** Steven R. Heidemann, Dennis Bray

**Affiliations:** ^1^Department of Physiology, Michigan State UniversityEast Lansing, MI, USA; ^2^Department of Physiology, Development and Neuroscience, University of CambridgeCambridge, UK

**Keywords:** axonal elongation, biomechanics, cytoskeleton dynamics, neural development, mechanotransduction, cellular

As the contents of this issue of “Frontiers” attest, the study of the mechanical aspects of neuronal development has come a long way since the 1970's and 80's when we became interested in the topic. The vast majority of cells in multicellular organisms are continually pushed and pulled, compressed, and stretched throughout their life and a diversity of mechanisms have evolved to protect against and harness these forces. Nerve cells are no exception, and several excellent reviews in recent years have examined ways in which mechanical inputs influence their development and function (Smith, 2009; Suter and Miller, 2011; Franze, 2013). However, it seems to us that certain aspects of this response have not been given the attention they deserve. In particular tension-driven axon assembly seems to be in a class of its own and quite distinct from other effects of tension such as modifications of cell migration, perturbation of division cycles, or changes in synthetic activity. Axon growth under these conditions is an exaggerated transformation of a nerve cell manifest by the prolonged accretion of new cellular material. Under the right circumstances, the rate and extent of this mass addition is remarkably large, to the degree that it implies an unusual, possibly unique mechanism. A pulled axon grows as though the nerve cell contained telescopic machinery prefabricated for elongation. But the identity of this nascent structure (if it exists), where it is stored, and how it is triggered to self-assemble into axon, remain to be discovered.

Mature axons have been known to be under tension since the early days of neuroanatomy. Harrison described the second phase of growth in which axons, having reached their target tissue while the axon is very short, increase their length coordinate with surrounding tissues—referring to it as “passive stretching” (Harrison, 1935). Weiss (1941) explicitly postulated that growth following synaptogenesis was due to mechanical tension. He described the “towing” of the axon as a result of the migration of the post-synaptic cell, as in the lateral line organ of zebrafish (Gilmour et al., 2004). Much later it became evident that mechanical tension has an essential role in the other, first phase of growth. Growth cones from individual sympathetic neurons growing in culture were recorded migrating away from the cell soma, pulling out neurites as they went (Bray, 1979). Vectorial analysis of the outgrowths produced in this way confirmed that they were tension-generated networks anchored at their free ends.

Incidentally, the curious observation was made in this early work that growth cones could be redirected by displacing their neurites with a fine microneedle. Pulling the neurite in a southwesterly direction, for example, caused the growth cone to head northeast, and so on. Growth was always along the vector of maximum tension and, even more dramatically, removal of the tension on a neurite caused the growth cone to bifurcate to produce a branch. This effect is as yet unexplained but seems to imply that tension within the body of the growth cone can direct the assembly of cytoskeletal structures such as microtubules and filopodia.

A series of experiments performed in the 1980s showed that, under appropriate conditions, mechanical tension is the determining stimulus leading to formation and elongation of an axon. Growth cones, or more precisely the terminal segment of neurites, were lifted from the culture surface by means of an electrode and then pulled under carefully controlled conditions. The axons formed in these experiments had a normal diameter, contained a typical cytoskeletal array, and were capable of subsequent elongation via a growth cone. Growth cones and axons could also be initiated *de novo* from embryonic chick sensory and forebrain neurons (Bray, 1984; Chada et al., 1997). In embryonic rat hippocampal neurons, applied tension was shown to specify which of several initial neurites take on a differentiated axonal fate. In a neuron typically extending only one axon, tension could stimulate formation of multiple axons (Lamoureux et al., 2002).

This approach has shown that all tested axonal types grow in response to applied tension and, particularly significant, the rate of elongation is directly proportional to the magnitude of applied tension. This robust linear function (*r* > 0.9 in 97% of trials) has been demonstrated for embryonic chick sensory (Zheng et al., 1991) and forebrain neurons (Chada et al., 1997) as well as embryonic rat hippocampal neurons (Lamoureux et al., 2002), and rat retinal ganglion neurons (Steketee et al., 2014). Such simple proportionality between tensions and elongation rates is the defining characteristic of an ideal (Newtonian) fluid-mechanical element, a dashpot. That is, the experimenter (or growth cone) produces a pulling force that the axon accommodates in a fluid-like manner, dissipating the force by elongating more axon. This is a strong physical argument for an immediate and direct relationship between tension and the axonal assembly process (O'Toole et al., 2008).

Experimentally applied mechanical tension can cause far more robust axonal growth than is observed “physiologically,” either *in vitro* or *in situ*. The most extreme example is the work of Smith and Pfister (Pfister et al., 2004; Smith, 2009). Embryonic (E15) rat dorsal root ganglia were explanted onto two overlapping membranes that were then gradually separated by a stepper motor, i.e., placed under continuous mechanical tension, such that the axons were elongating in the space between the membranes. This experimental system supported axonal elongation rates of ~400 μm/h, ~10-fold greater than the typical advance rate of growth cones! As for mass addition, this protocol permitted axonal tracts some 10 cm in length after 2 weeks of towing with axonal diameters 30% greater than cultures grown out solely by growth cone activity. The axons elongated by experimental tension were shown to have a normal cytoskeletal array and were electrically active (Pfister et al., 2006, see also Loverde and Pfister, 2015, this issue).

The data are persuasive therefore that tension can be the proximate cause for axonal elongation. By “proximate” we mean a situation in which mechanical tension is the immediate stimulus for axonal elongation, and apparently axonal initiation and specification as well. Clearly myriad longer-time-scale regulators are also necessary, such as appropriate trophic factors, growth-associated proteins, and specializations of the cytoskeleton. But with all that in place, it is undeniable that there are situations in which mechanical tension is the determining stimulus leading to formation and elongation of an axon. But how does this work? What is it in an axon that the mechanical signal acts upon and how is this initial response transduced into a cascade of biochemical and cellular changes? The conditions for the various experimental “towing” interventions apparently disqualify the typical mechanotransduction pathways. In particular, axons are suspended in the culture medium during most protocols to experimentally elongate axons, i.e., the axon is largely isolated during towing. This argues that the tension-sensing mechanism is entirely contained within the axon. So, for example, adhesions or changes of adhesion to a substrate, probably the most widely cited subprocess of cellular mechanotransduction (Ingber, 2006; Hoffman et al., 2011; Iskratsch et al., 2014), apparently do not directly contribute to tension-induced mass addition. Similarly, there are no changes in cellular motility; no changes in cell–cell interactions; and no changes in cell stiffness (again, a Newtonian dashpot). These “disqualifications” highlight the possibly unique status of neuronal elongation in response to tension. We can only speculate on the possible mechanism.

From a purely geometric standpoint, a nerve cell that produces 1 mm of axon in a day (a typical rate in a towing experiment) increases its length by several orders of magnitude more than its radius making longitudinal addition of material the dominant factor in response to pulling. Thus, longitudinal elements such as microtubules, intermediate filaments, and actin filaments, should be selectively up-regulated in a towed neuron, to a much greater degree than components of the cytosol or nucleus. Moreover, since the bulk of this synthesis will take place in the cell soma, we also anticipate a major increase in the transport of these proteins and their assembly at the site of growth.

Providentially, recent findings concerning the transport and assembly of cytoskeletal elements in an axon seem to offer a possible mechanism (Figure 1). At steady state (Figure 1A), short segments of both microtubules and neurofilaments have been observed to move rapidly within the axon in either direction, driven by molecular motors, and to be able to add to more static structures (Wang and Brown, 2002; Baas et al., 2006; Brown and Jung, 2013). These motile segments therefore offer a potential source for mass addition, available at short notice at any point along the axon. Conceivably they could even underpin the notional telescopic mechanism for elongation mentioned above. But how could tension cause mobile segments of microtubules and neurofilaments to accumulate at the right location and when required?

**Figure 1:**
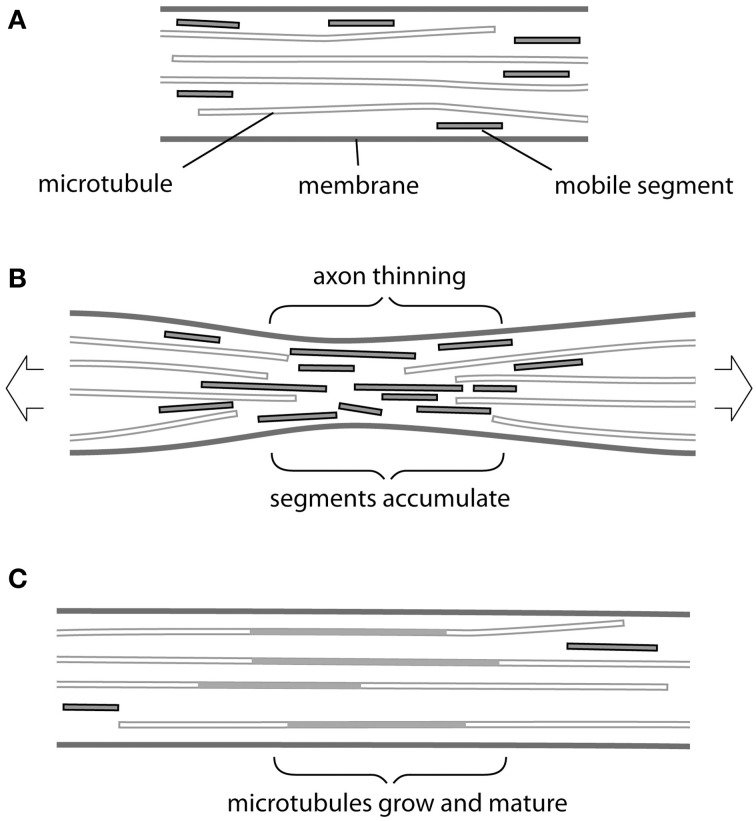
**Possible mechanism of tension-driven axon growth. (A)** Region of axon is shown containing long and short microtubules. Short microtubule segments (red) are rapidly transported in both anterograde and retrograde direction by molecular motors. Note that neurofilaments, which behave in a similar fashion, are omitted for clarity. **(B)** Tension is created in the axon by pulling. This causes the axon to become thinner and long microtubules to be drawn apart longitudinally. At the same time thinning causes microtubules to be pushed together laterally. Rapid transport is no longer possible across this region and mobile segments build up in a traffic jam. **(C)** The accumulated microtubule segments rearrange and add to free ends. Eventually new lengths of microtubule become stabilized by associated proteins, causing their lateral spacing to increase and the axonal diameter to return to its original value.

The simplest answer seems to us to be thinning of the axon during stretch (Figure 1B). Here, we are focused on stretch that does not cause evidence of injury (Loverde and Pfister, 2015, this issue). Although in such cases the final diameter of axons produced by towing is reported to be normal, in every series of experiments there are instances in which thinning and eventual breakage of neurites occurs. This was especially noticeable in axons towed rapidly (Fass and Odde, 2003) and—interestingly—also conspicuous following treatment with the microtubule poison vinblastine (Zheng et al., 1993). If we consider that, even in the hands of the most skilled operator, some degree of thinning must occur then we have a plausible first step for our response. Tension will draw microtubules and neurofilaments apart longitudinally and possibly also cause them to break (Tang-Schomer et al., 2012). At the same time these cytoskeletal elements will be forced closer together by the thinning of the axon, so that the space between them available for transport will be reduced. It may be noted that, compaction of microtubules and neurofilaments was directly observed in thinned regions of axons following stretching and fast-freezing (Ochs et al., 1997). Breakage and compaction conspire to impede the progress of shorter more mobile segments of microtubules and neurofilaments, which will therefore pile up at the site of constriction in a “traffic jam” (Figure 1B). We can then imagine the normal process of maturation taking place (Figure 1C) in which the accumulated segments add to the cytoskeletal framework of the axon and acquire a complement of associated proteins, such as the newly described complex between ankyrin and MAP1B (Stephan et al., 2015). Spaces between microtubules and neurofilaments will be restored and new channels created. Transport will resume and the original axon diameter will return to its original value…at this point tension will have caused the axon to grow.

Clearly this description omits many essential steps. Up-regulation of the synthesis of tubulin and neurofilament protein must occur in the cell body together with that of actin filaments and membrane components such as channels. These all have to be transported into the axon and assembled in the correct location. But the notion of an accumulation of cytoskeletal and membrane components triggered by axon thinning and their subsequent rearrangement and maturation could explain the remarkable and possibly unique response of neurons to tension.

## Conflict of interest statement

The authors declare that the research was conducted in the absence of any commercial or financial relationships that could be construed as a potential conflict of interest.
